# Preparation and Characterization of Thermo-Compressed Guar Gum/Microcrystalline Cellulose Composites for Applications in Sustainable Packaging

**DOI:** 10.3390/polym17233124

**Published:** 2025-11-25

**Authors:** Prasong Srihanam, Jenjira Jirum, Pakin Noppawan, Nuanchai Khotsaeng, Yodthong Baimark

**Affiliations:** 1 Biodegradable Polymers Research Unit, Department of Chemistry and Centre of Excellence for Innovation in Chemistry, Faculty of Science, Mahasarakham University, Maha Sarakham 44150, Thailand; jenjira.j@msu.ac.th; 2 Department of Chemistry and Centre of Excellence for Innovation in Chemistry (PERCH-CIC), Faculty of Science, Mahasarakham University, Maha Sarakham 44150, Thailand; pakin.n@msu.ac.th; 3 Sustainable Approaches for Materials, Agriculture, and Health Technology (SAMAHT) Research Unit, Mahasarakham University, Maha Sarakham 44150, Thailand; 4 Faculty of Science and Health Technology, Kalasin University, Namon District, Kalasin 46230, Thailand; nuanchai.k@ksu.ac.th

**Keywords:** guar gum, glycerol, microcrystalline cellulose, plasticization, reinforcement, thermo-compression

## Abstract

In this study, we prepared guar gum (GG) films using a compression molding technique for the first time, incorporating glycerol as a plasticizer and microcrystalline cellulose (MCC) as a reinforcing filler. The chemical structures, thermal properties, crystalline structures, phase morphology, mechanical properties, moisture content, and film opacity of thermo-compressed GG films were investigated. The results show that using glycerol as a plasticizer enhanced the flexibility of the thermo-compressed GG film and promoted its crystallization. The incorporation of glycerol enhanced the thermal stability of the GG film matrix. The addition of MCC enhanced the tensile strength of the plasticized GG film; however, it resulted in a decrease in elongation at break. The incorporation of MCC in plasticized GG films resulted in enhanced opacity and a decrease in moisture content. Thermo-compressed GG films can be customized to exhibit various properties by adjusting the glycerol and MCC contents, making them suitable for a range of eco-friendly and sustainable packaging applications.

## 1. Introduction

Plastic waste is a significant global pollution issue, especially concerning single-use plastic packaging. Plastic waste like polypropylene, polyethylene, and polystyrene can take hundreds of years to decompose [1]. The idea of utilizing biodegradable polymers for different types of plastic packaging has emerged as a potential solution to replace conventional plastics and mitigate pollution caused by plastic waste. Biodegradable polymers are those that can be degraded through simple hydrolysis reactions, and the products can be further degraded by microorganisms [2,3]. Biodegradable polymers can be categorized into two main types: petroleum-based polymers and bio-based polymers. Petroleum-based polymers include poly(ε-caprolactone) (PCL), poly(butylene adipate-*co*-terephthalate) (PBAT), and poly(butylene succinate) (PBS). In contrast, bio-based polymers or biopolymers consist of poly(lactic acid) (PLA), polyhydroxyalkanoates (PHAs), and natural materials, which include polysaccharides (such as starch, cellulose, chitosan, guar gum, agar, alginate, and carrageenan), as well as proteins (including silk sericin, silk fibroin, and keratin). Biopolymers have a lower carbon footprint compared to their petroleum-based counterparts [1,4,5,6]. The European Bioplastics Association defines biopolymers as materials that are either bio-based, biodegradable, or both [7]. Currently, numerous reports highlight research and development efforts that focus on biopolymers for packaging applications [4,6,8]. This innovative material is being explored not only for its environmental benefits but also for its potential to meet the growing demand for eco-friendly and sustainable packaging solutions. As research advances, researchers are optimizing the performance characteristics of these polymers to improve their suitability for commercial packaging applications.

Guar gum (GG) is a high-molecular-weight polysaccharide that is specifically classified as a polymer of galactomannan. It is composed of mannose sugar molecules linked by *β*-1,4-glycosidic bonds, with galactose sugar branches connected by *α*-1,6-bonds. GG has been widely studied and developed for various applications, including biomedical [9,10], the food industry [9], wastewater treatment [9,11], cosmetics [9], the petroleum industry [12], and packaging [9,13,14]. GG is a hydrocolloid that has garnered significant attention for its potential as a food packaging material, primarily due to its good film-forming ability, non-toxicity, biodegradability, low cost, and widespread availability [13,14,15]. However, in the past, industrial production has encountered considerable limitations because it has relied almost exclusively on the solution casting technique for the formation of GG-based packaging products. To the best of our knowledge, there have been scarcely any reports on the formation of GG-based films using melt processing techniques [15]. This research is the first report to document the production of GG films using the compression molding technique.

Polysaccharide films typically contain glycerol as a plasticizer to improve their flexibility. Before casting, glycerol is incorporated into GG films by mixing it with the GG solution [16,17,18,19,20]. Microcrystalline cellulose (MCC) is a natural filler derived from agricultural waste materials [21,22]. It plays an active role in the formulation of various polysaccharide films, enhancing their strength [23,24,25,26]. When external forces apply, the hydrogen bonding between microcrystalline cellulose (MCC) and the polysaccharide facilitates effective stress transfer. This study employed glycerol as a plasticizer and microcrystalline cellulose (MCC) as a reinforcing filler in the formulation of thermo-compressed GG films. We hypothesized that glycerol and microcrystalline cellulose (MCC) would influence the flexibility and strength of the thermo-compressed GG films. To evaluate this hypothesis, we conducted two sample series (glycerol-plasticized GG films and MCC-reinforced/glycerol-plasticized GG films) of tensile tests. We also determined the chemical structures, phase morphology, thermal stability, crystalline structures, moisture content, and opacity of the thermo-compressed GG films.

## 2. Materials and Methods

### 2.1. Materials

Food-grade guar gum (GG) powder with 82% min galactomannan content and a viscosity of 6000 cps (1% aqueous solution) was purchased from Chanjao Longevity Co., Ltd. (Bangkok, Thailand). Microcrystalline cellulose (MCC) with an average particle size of 50 µm was supplied by Acros Organics (Ward Hill, MA, USA). Glycerol (99.5%) was obtained from QReC (Pathum Thani, Thailand).

### 2.2. Preparation of Thermo-Compressed GG-Based Films

**Figure 1:**
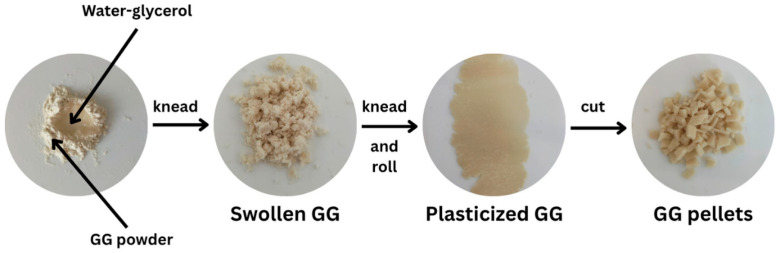
Preparation of glycerol-plasticized GG pellets.

To study the plasticization effectiveness of glycerol on GG film, GG powder (10 g) was mixed with a glycerol aqueous solution (30 g) and kneaded until a homogeneous mixture was achieved. The glycerol aqueous solution was prepared by dissolving an appropriate amount of glycerol in water. The mixture was then rolled and cut into pellets with scissors, as illustrated in Figure 1. We investigated glycerol contents of 0, 15, 30, and 45 wt% based on the weight of GG. The plasticized GG pellets were subjected to thermo-compression at 120 °C for a duration of 5 minutes, applying a force of 5 MPa using an Auto CH Carver hot-press machine (Wabash, IN, USA). The films were then cooled using cool plates while maintaining a compressed force of 5 MPa for 5 minutes. The films were fixed on plastic mesh frames and subsequently dried in an airflow oven at 30 °C for a duration of 6 hours. Before characterization, the films were stored at room temperature (25–30 °C) and at a relative humidity (RH) of 50–60% for 14 days [27,28]. A glycerol-free GG film was also prepared under the same conditions with a mixture of GG powder (10 g) and distilled water (30 g) for comparison.

To evaluate the reinforcement effectiveness of MCC on glycerol-plasticized GG film, the mixture of GG and MCC was kneaded and rolled with a glycerol aqueous solution, following the same method described previously. We maintained a constant glycerol content of 30 wt%, which was calculated based on the weight of GG. We examined the MCC contents of 5, 10, 20, and 30 wt% based on the weight of GG. The glycerol-plasticized GG pellets mixed with MCC were hot-pressed and then dried at 30 °C in an airflow oven for 6 hours before being stored for 14 days using the same methods described earlier before characterization.

### 2.3. Characterization of GG Powder, MCC Powder, and Thermo-Compressed GG Films

#### 2.3.1. FTIR Analysis

A Fourier transform infrared (FTIR) spectrometer equipped with attenuated total reflection (ATR) diamond (Invenio-S, Bruker, Karlsruhe, Germany) was used to analyze the chemical structures of each sample. The ATR-FTIR spectra were collected at a wavenumber range of 500–4000 cm^−1^ with an accumulation of 32 scans and a resolution of 4 cm^−1^.

#### 2.3.2. Thermal Stability

The thermal stability of each sample (~10 mg) was analyzed with a thermogravimetric analyzer (TGA, SDT Q600, TA-Instruments, New Castle, DE, USA) under nitrogen flow at a rate of 100 mL·min^−1^. All the samples were scanned from 50 to 800 °C at a heating rate of 20 °C·min^−1^.

#### 2.3.3. Crystalline Structures

X-ray diffraction (XRD) analysis was used to determine the crystalline structures of each sample using an X-ray diffractometer (D8 Advance, Bruker, Karlsruhe, Germany) with a CuKα source operated at 40 kV and 40 mA. The XRD pattern was recorded from 5 to 60° of 2θ diffraction angle, and the scan rate was 3° min^−1^.

#### 2.3.4. Mechanical Testing

Tensile tests were performed to determine the mechanical properties of the film samples (60 mm × 10 mm) using a universal testing machine (LY-1066B, Dongguan Liyi Environmental Technology Co., Ltd., Dongguan, China) with a 100 kg load cell at 25 °C. The test was performed with an initial gauge length of 40 mm and a crosshead speed of 50 mm·min^−1^ For each sample, five films were evaluated, and both the average values and the standard deviation were recorded.

#### 2.3.5. Morphology Analysis

The cryo-fractured surfaces of the film samples prepared in liquid nitrogen were examined with a scanning electron microscope (SEM, JSM-6460LV, JEOL, Tokyo, Japan) at 15 kV. All samples were sputtered with gold before SEM analysis.

#### 2.3.6. Moisture Content

The film samples (20 mm × 20 mm) were weighed (*W*_1_) before drying at 105 °C for 24 h. The film samples were weighed again (*W*_2_) after drying. The moisture content of the film samples was calculated using the following equation. Each sample was tested on three films, and the average ± standard deviation values were given.

Moisture content (%) = [(*W*_1_ − *W*_2_)/*W*_1_] × 100 (1)

#### 2.3.7. Film Opacity

Film opacity of the film samples was determined from absorbance at a wavelength of 600 nm (*A*_600_) using a UV-Vis spectrophotometer (Cary 60, Agilent Technologies, Victoria, Australia). The film opacity was calculated using the following equation [29,30]. Three measurements were taken for each sample, and the mean and standard deviation values were reported.

Film opacity (mm^−1^) = *A*_600_/*X* (2)

where *X* is the thickness of the film sample (mm).

### 2.4. Statistical Analysis

The experimental data were analyzed using one-way ANOVA, followed by Duncan’s post hoc test. The results are expressed as the mean ± standard deviation (SD). Statistical analyses were conducted using SPSS version 22.0, with statistical significance established at *p* < 0.05 for all experiments.

## 3. Results and Discussion

### 3.1. Effect of Glycerol on Properties of GG Films

#### 3.1.1. FTIR Analysis

The chemical functional groups present in the films, as well as the potential intermolecular interactions among the film components (GG, water, and glycerol), were analyzed using ATR-FTIR spectra. An ATR-FTIR spectrum of GG powder, as shown in Figure S1a, reveals a broad band at 3283 cm^−1^ corresponding to O–H stretching vibrations and adsorbed water molecules in the film [15,20]. A band at 2883 cm^−1^ shows that C–H stretching vibrations are present [20,26]. Additionally, a band at 1639 cm^−1^ represents the ring stretching of GG and O–H bending associated with water [31,32]. The spectrum also shows a band at 1376 cm^−1^ for C–H bending vibrations and a sharp band at 1016 cm^−1^ for O–H bending vibrations [31], as well as C–O–C stretching vibrations from the glycosidic bonds of GG [26,33]. Furthermore, a band at 869 cm^−1^ corresponds to mannose and galactose 1–4 and 1–6 linkages in GG molecules [18,33], and a band at 810 cm^−1^ indicates glycosidic linkages from galactopyranose units in GG molecules [33,34].

**Figure 2:**
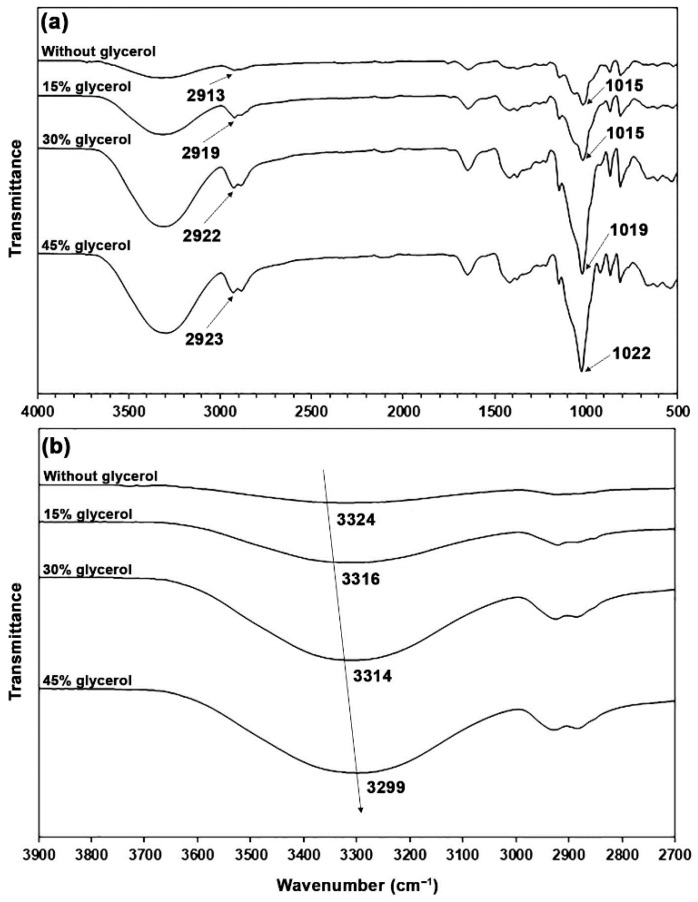
(**a**) ATR-FTIR spectra and (**b**) expanded hydroxyl group regions of glycerol-plasticized GG films with varying glycerol content.

The thermo-compressed GG films with and without glycerol showed the same ATR-FTIR pattern as the GG powder, as shown in Figure 2a. However, differences in the intensity of bands could have occurred. The band intensities of O–H stretching vibration in the range of 3000–3700 cm^−1^ increased with the glycerol content as a result of the increasing hydroxyl groups provided by glycerol. The band intensities in the range of 800–1150 cm^−1^ attributed to glycerol molecules also increased [18]. In Figure 2b, the O–H band of a glycerol-free GG film at 3324 cm^−1^ shifted to lower wavenumbers when glycerol plasticizer was incorporated (3316, 3314, and 3299 cm^−1^ for 15, 30, and 45 wt% glycerol, respectively), which represents the hydrogen-bonding interactions among the hydroxyl groups of GG, absorbed water, and glycerol [35,36].

The spectrum of a glycerol-free GG film exhibited a band at 2913 cm^−1^, indicative of the asymmetric C–H stretching vibration of methylene groups. It was observed at 2919, 2922, and 2923 cm^−1^ when glycerol was present in concentrations of 15, 30, and 45 wt%, respectively. The observed shift toward higher wavenumbers suggests that there are weaker interactions with the plasticizers [35]. A band at 1015 cm^−1^ in a glycerol-free GG film is associated with O–H bending vibrations [31] and C–O–C stretching vibration linkages from the glycosidic bonds of GG [26,33]. Arfat et al. [37] attribute this band to interactions between the film matrix and the absorbed water. The band was detected at 1015 cm^−1^ for a glycerol content of 15 wt%, at 1019 cm^−1^ for 30 wt%, and at 1022 cm^−1^ for 45 wt%, respectively. The shift toward higher wavenumbers, which indicates hydrogen bonding between absorbed water and GG, was diminished due to the increased affinity of water for glycerol [28,38].

#### 3.1.2. TGA

We studied the effect of glycerol (Gly) plasticization on the thermal decomposition behaviors of GG films using thermogravimetric (TG) and derivative TG (DTG) thermograms in the temperature range of 50–800 °C. The TG and DTG thermograms of GG powder in Figure S2a demonstrated two major steps of weight loss: the first weight-loss step in the temperature range of 50–120 °C is related to the evaporation of residue moisture, and the second weight-loss step in the temperature range of 250–400 °C corresponds to the thermal decomposition of GG [20,39]. The char residue at 800 °C in the GG powder was 16.4%. The maximum decomposition temperature of the GG fraction (*GG-T_max_*) obtained from the DTG thermogram was 311 °C. This value is similar to that found in previous work [39].

**Figure 3:**
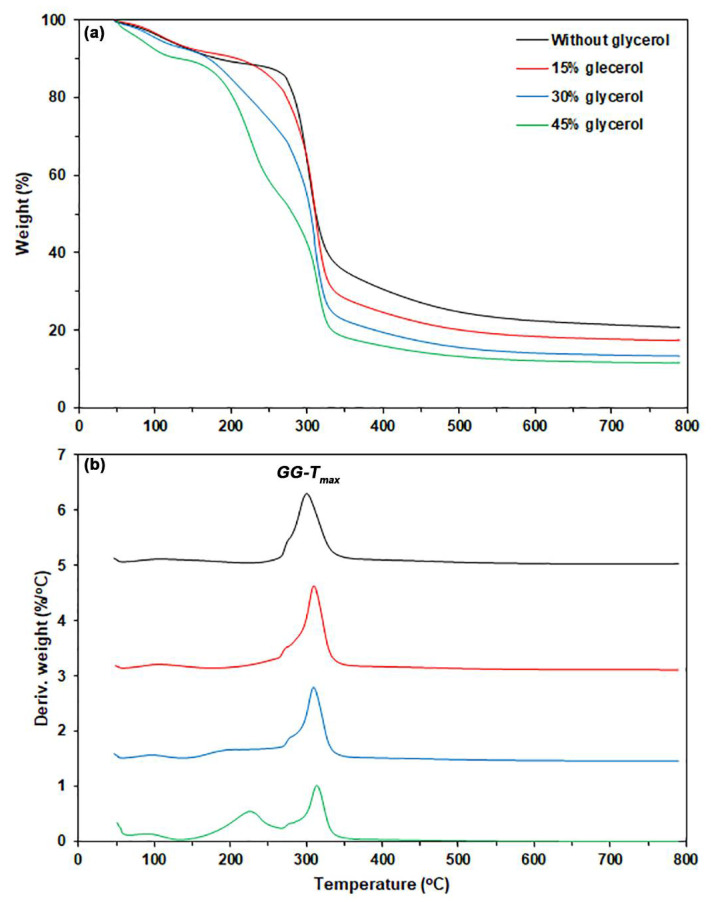
(**a**) TG and (**b**) DTG thermograms of glycerol-plasticized GG films with varying glycerol content.

Figure 3 presents the TG and DTG thermograms, as shown in Figure 3a and Figure 3b respectively, for both the pure GG and glycerol-plasticized GG films, with a summary of the TGA results provided in Table 1. The pure GG film exhibited two major steps of weight loss. The first weight-loss step (5.5%) was in the temperature range of 50–120 °C, and the second weight-loss step was in the temperature range of 250–400 °C, as shown in Figure 3a. The char residue at 800 °C of the pure GG film was 20.8%. The *GG-T_max_* of pure GG film was 300 °C. This observation suggests that the thermal decomposition of pure GG film was faster than that of GG powder. This effect may be due to some GG molecules thermally decomposing during the compression molding process.

The DTG curves in Figure 3b for glycerol-plasticized GG films displayed a new DTG peak in the temperature range of 120–260 °C, which is attributed to the evaporation of glycerol [40]. The height of this DTG peak increased with the glycerol content. The moisture weight loss of glycerol-plasticized GG films, as reported in Table 1, increased with the glycerol content. Films with more glycerol in them were more hydrophilic, which helped them absorb more moisture during the 14-day storage period. As the glycerol content increased, the char residue at 800 °C in the film samples steadily decreased due to a reduction in the GG content of the films. The *GG-T_max_* values of glycerol-plasticized GG films (310–314 °C) were higher than the pure GG film (300 °C), suggesting that the added glycerol improved the thermal stability of the GG film matrix.

#### 3.1.3. XRD

**Figure 4:**
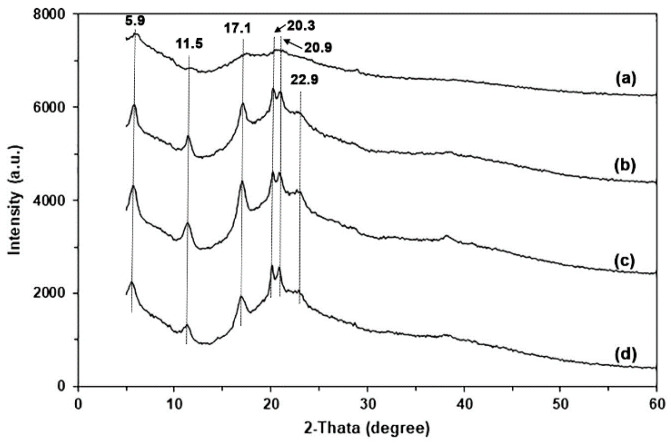
XRD patterns of (**a**) pure GG film and glycerol-plasticized GG films with glycerol contents of (**b**) 15 wt%, (**c**) 30 wt%, and (**d**) 45 wt%.

The XRD pattern of GG powder shown in Figure S3a indicated low overall crystallinity, featuring a small diffraction peak corresponding to the crystalline form of native GG at 2θ = 20.3° [33,41]. Figure 4 displays the XRD patterns of GG films, comparing those with glycerol to those without. The glycerol-free GG film exhibited a prominent XRD peak at 2θ = 20.3°, along with smaller XRD peaks at 2θ = 5.9°, 11.5°, and 17.1°. These peaks were more pronounced when glycerol was added. This behavior suggests that the addition of glycerol resulted in an enhanced ordered phase. Furthermore, we noted an increase in the intensities of the XRD peaks at 2θ = 20.3°, 20.9°, and 22.9°. The XRD peaks observed at 5.9° correspond to B-type crystallinity, and 17.1° and 22.9° correspond to A-type crystallinity of polysaccharides [25,42]. The presence of water (a de-structuring agent) and glycerol (a non-volatile plasticizer) during compression molding may enhance the chain mobility of GG and improve the arrangement and packing of GG molecules, resulting in a more compact organization within the ordered domains [43]. The XRD results suggest that glycerol enhanced the formation of crystalline structures within the GG film matrix.

#### 3.1.4. Tensile Properties

**Figure 5:**
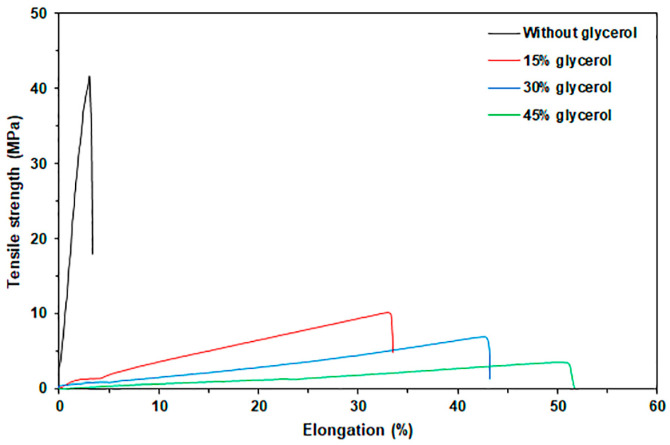
Selected tensile curves of glycerol-plasticized GG films with varying glycerol content.

Figure 5 displays selected tensile curves for pure GG and glycerol-plasticized GG films, while Table 2 provides a summary of the tensile results. The pure GG film exhibited a maximum tensile strength of 40.6 MPa, an elongation at break of 3.4%, and a Young’s modulus of 988.2 MPa. The incorporation of glycerol led to a significant decrease in both the maximum tensile strength and Young’s modulus values, while the elongation at break increased dramatically. This finding suggests that glycerol acts as an effective plasticizer for the GG. Glycerol is a common plasticizer for polysaccharides because it enhances their chain mobility by reducing intermolecular forces between polymer chains, making them more flexible and less rigid [44]. Various starch films also demonstrated this behavior [45,46,47,48]. In addition, Jiang et al. [13] reviewed studies indicating that glycerol is the most effective plasticizer for GG films produced using the solvent casting method.

As the glycerol content increased, there was a consistent decrease in both the maximum tensile strength and Young’s modulus values, along with a significant increase in elongation at break. This trend can be attributed to glycerol’s role in reducing the strong intramolecular forces among polysaccharide chains while also promoting the formation of hydrogen bonds between glycerol and polysaccharide molecules. The reduction in tensile strength of plasticized polysaccharide films can be attributed to the diminished hydrogen bonds among the polysaccharide chains [49]. Furthermore, the increased moisture absorption related to higher glycerol content, as indicated in Table 1, resulted in a reduction of the maximum tensile strength of GG films while concurrently enhancing the elongation at break. This outcome occurs because the moisture molecules that are absorbed function as plasticizers [50]. Based on the tensile results, it can be concluded that glycerol improved the flexibility of thermo-compressed GG films. 

#### 3.1.5. SEM

**Figure 6:**
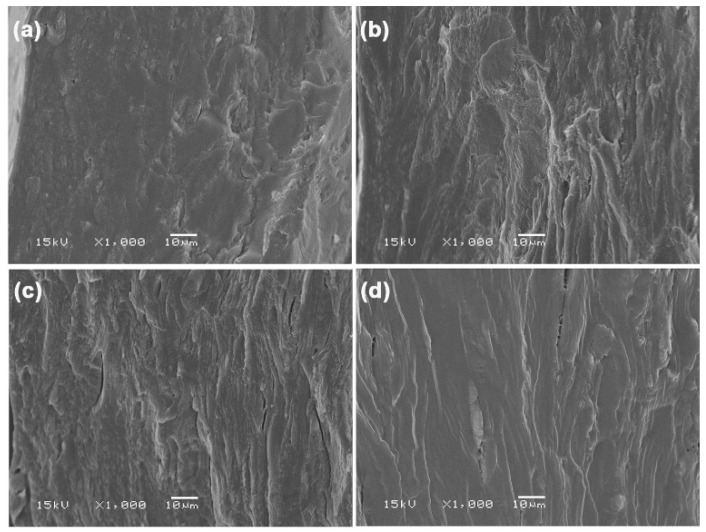
SEM images of cryogenically fractured surfaces of (**a**) pure GG film and glycerol-plasticized GG films with glycerol contents of (**b**) 15 wt%, (**c**) 30 wt%, and (**d**) 45 wt%.

The phase morphology of the plasticized GG films was examined using SEM images of their cryogenically fractured surfaces, as illustrated in Figure 6. The absence of visible GG particles in these film matrices indicates that the kneading and rolling process used in this work effectively plasticizes the GG particles. The pure GG film exhibits the smoothest surface texture, suggesting that it is the most brittle, which aligns with the previously mentioned tensile results. The lack of sufficient plasticizer distribution, crucial for enhancing the material’s flexibility, may be the cause of this brittleness. The observed morphology of the fractured surface is directly linked to the mechanical properties, emphasizing the relationship between the film’s structure and its performance. The fractured surface texture of the plasticized GG films appears rougher, suggesting that they possess greater flexibility compared to pure GG film. The observed rough cross-section structures may be due to insufficient interfacial adhesion between the GG film matrix and glycerol plasticizer, leading to weak forces during the tensile test and a subsequent decrease in tensile strength and increase in elongation at break [49].

Additionally, we observed that the incorporation of 45% glycerol in Figure 6d led to greater homogeneity compared to the 15% and 30% glycerol concentrations shown in Figure 6b and Figure 6c respectively. A similar observation indicated that the cross-section of biopolymer films with higher glycerol content demonstrated increased homogeneity [51]. The findings suggested that films with higher glycerol content displayed greater homogeneity compared to those with lower glycerol content.

#### 3.1.6. Moisture Content and Film Opacity

**Figure 7:**
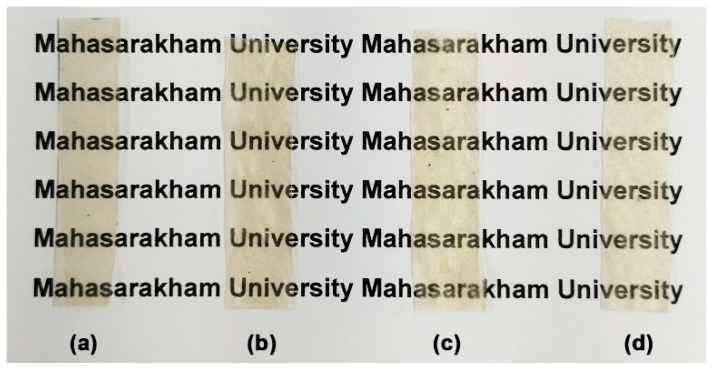
Photographs of thermo-compressed films of (**a**) pure GG and glycerol-plasticized GG with glycerol contents of (**b**) 15 wt%, (**c**) 30 wt%, and (**d**) 45 wt%.

Table 3 demonstrates the influence of glycerol content on the thickness, moisture content, and opacity of GG films. All films exhibited thicknesses between 0.19 and 0.21 mm, with opacity values ranging from 1.67 to 1.71 mm^−1^. The incorporation of glycerol did not influence the thickness and opacity of GG films. The moisture content of GG films significantly increased as the glycerol content rose, which is attributed to glycerol’s highly hydrophilic nature. Various hydrocolloid films also demonstrated this behavior [52,53,54]. Figure 7 displays the characteristics of pure GG film alongside those that have been plasticized with glycerol. All films display transparency, providing a clear view of the characters beneath them.

### 3.2. Effect of MCC on Properties of Plasticized GG Films

#### 3.2.1. FTIR Analysis

The ATR-FTIR spectrum of MCC in Figure S1b displays several characteristic bands. It shows a broad band at 3329 cm^−1^ corresponding to O–H stretching vibrations, a band at 2886 cm^−1^ associated with C–H stretching vibrations, and a band at 1426 cm^−1^ indicative of intermolecular hydrogen bonding at C6 of the aromatic ring groups. Additionally, there is a band at 1162 cm^−1^ related to C–O–C stretching vibrations of the *β*-1,4-glycosidic linkage [55]. A band at 1076 cm^−1^ corresponds to the deformation of the glucopyranose ring [56]. Furthermore, bands at 1035 and 1030 cm^−1^ are attributed to C–O stretching vibrations of cellulose [57], while a band at 895 cm^−1^ is associated with the *β*-1,4-glycosidic linkage [58].

**Figure 8:**
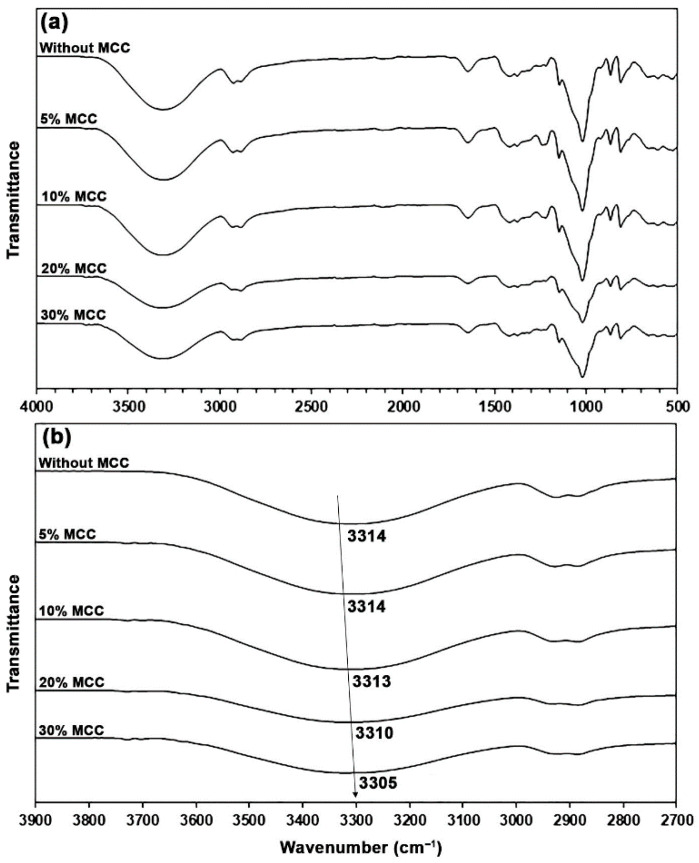
(**a**) ATR-FTIR spectra and (**b**) expanded hydroxyl regions of MCC-reinforced and glycerol-plasticized GG films with varying MCC content. All films contain 30 wt% glycerol.

In Figure 8a, the ATR-FTIR spectra of glycerol-plasticized GG films with MCC show a pattern similar to that of the glycerol-plasticized GG film without MCC. In Figure 8b, the band at 3314 cm^−1^ shifted to lower wavenumbers upon incorporating MCC, which corresponds to the O–H stretching vibration of a GG/30Gly film. The observed values were 3314, 3313, 3310, and 3305 cm^−1^ for 5, 10, 20, and 30 wt% MCC, respectively. This shift indicates that hydrogen-bonding interactions among the hydroxyl groups of GG, absorbed water, glycerol, and MCC occurred within the film matrix [35,36]. Previously, Tian et al. [59] reported a similar finding when they incorporated MCC into the starch film.

#### 3.2.2. TGA

**Figure 9:**
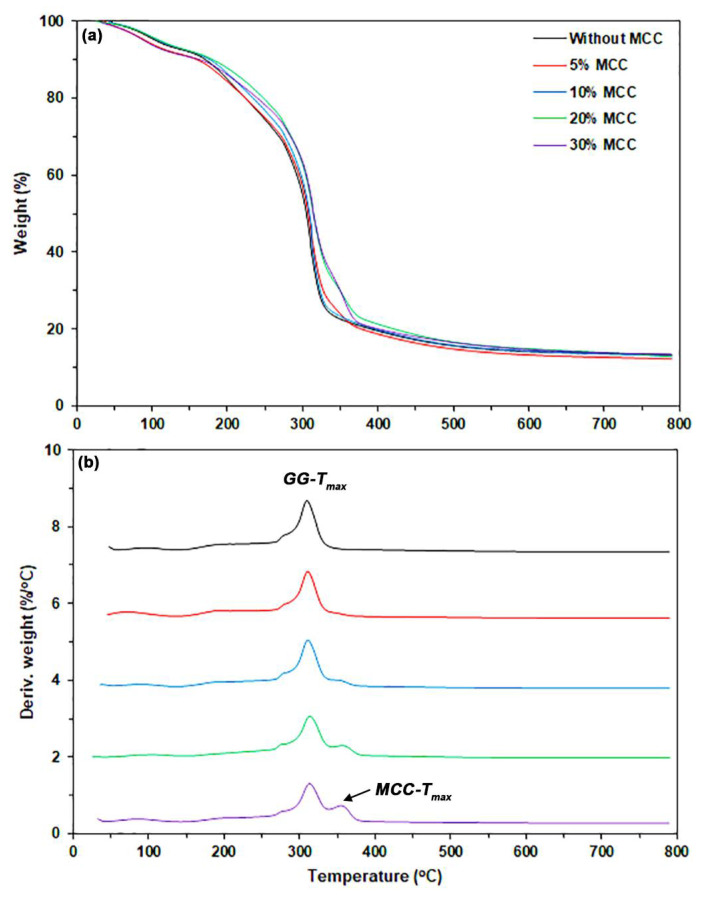
(**a**) TG and (**b**) DTG thermograms of MCC-reinforced and glycerol-plasticized GG films with varying MCC content. All films contain 30 wt% glycerol.

Figure 9a,b depict the TG and DTG thermograms, respectively, of MCC-reinforced and glycerol-plasticized GG films. Table 4 summarizes the TGA results of the MCC-reinforced and glycerol-plasticized GG films. The MCC-reinforced and glycerol-plasticized GG films had a char residue at 800 °C (12.9–13.3%) that was lower than the film without MCC (14.6%). This difference arises because the char residue of MCC at 800 °C is lower than that of GG (see Figure S2). According to Figure 9b, the *GG-T_max_* peaks of the film samples appear to shift to higher temperatures with the addition of MCC. The *GG-T_max_* peak of the glycerol-plasticized GG film without MCC was recorded at 310 °C. In contrast, the MCC-reinforced and glycerol-plasticized GG films exhibited *GG-T_max_* peaks ranging from 311 to 314 °C. The addition of MCC appears to slightly improve the thermal stability of the GG film matrix. As illustrated in Figure S2, the higher *GG-T_max_* of MCC (355 °C) accounts for the observed difference. The enhanced thermal stability of MCC functions as a barrier, limiting heat transfer through the GG matrix [60]. The temperature range of 355–358 °C reveals the *T_max_* peaks of MCC (*MCC-T_max_*) in the GG films that are reinforced with MCC and plasticized with glycerol.

#### 3.2.3. XRD

**Figure 10:**
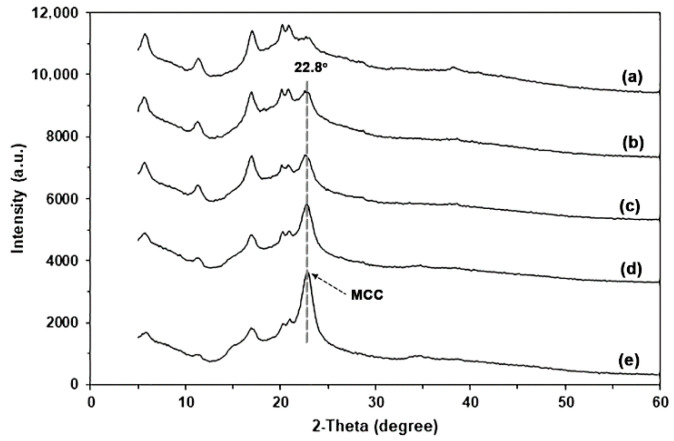
XRD patterns of glycerol-plasticized GG films: (**a**) without MCC and with MCC contents of (**b**) 5 wt%, (**c**) 10 wt%, (**d**) 20 wt%, and (**e**) 30 wt%. All films contain 30 wt% glycerol.

The XRD pattern of MCC, as shown in Figure S3b, exhibited peaks at 2θ = 15.2°, 22.8°, and 34.9°, which represent the crystalline characteristics of cellulose type I [61,62,63]. Figure 10 presents the XRD patterns of glycerol-plasticized GG films with and without MCC. The addition of MCC did not change the XRD patterns of the glycerol-plasticized GG film. The peak intensities of the characteristic features of the glycerol-plasticized GG film decrease with increasing MCC content. The XRD peak of MCC at 2θ = 22.8° demonstrated a steady increase in peak intensity, suggesting that GG films can be produced with different quantities of MCC.

#### 3.2.4. Tensile Properties

**Figure 11:**
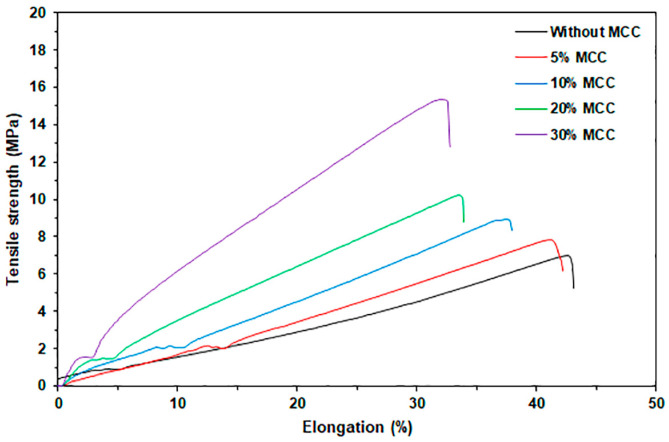
Selected tensile curves for MCC-reinforced and glycerol-plasticized GG films with varying MCC content. All films contain 30 wt% glycerol.

Figure 11 presents the tensile curves for MCC-reinforced and glycerol-plasticized GG films, while Table 5 provides a summary of the tensile results. The maximum tensile strength and Young’s modulus showed significant increases, while elongation at break exhibited a slight decrease as the MCC content rose. The tensile results demonstrated that the incorporation of MCC improved the reinforcement of the GG film matrix. Interactions between the GG and MCC may have facilitated effective stress transfer between the two materials, as described above in the FTIR analysis. A similar observation was made regarding the GG films produced using a solvent casting method, where enhanced mechanical properties were observed in GG films that incorporated MCC [26]. Moreover, several hydrophilic biopolymer films, including starch [27], thermoplastic starch (TPS) [23,25], and soy protein isolate [64], have employed MCC as a cost-effective reinforcing filler. MCC possesses the capability to serve as an efficient reinforcing agent for GG film while simultaneously reducing production costs.

This study found that the optimal amount of MCC was likely 30 wt%, resulting in a 100% increase in maximum tensile strength. However, this dosage also caused a 24% decrease in elongation at break when compared to the GG/30GLy film that did not contain MCC. However, the GG/30Gly/30MCC films exhibited lower maximum tensile strength (16 MPa) and elongation at break (32%) when compared to thermo-compressed polypropylene films, which demonstrated values of 38 MPa and 75%, respectively [65]. Therefore, further research and development are needed to improve the mechanical properties of these GG films.

#### 3.2.5. SEM

**Figure 12:**
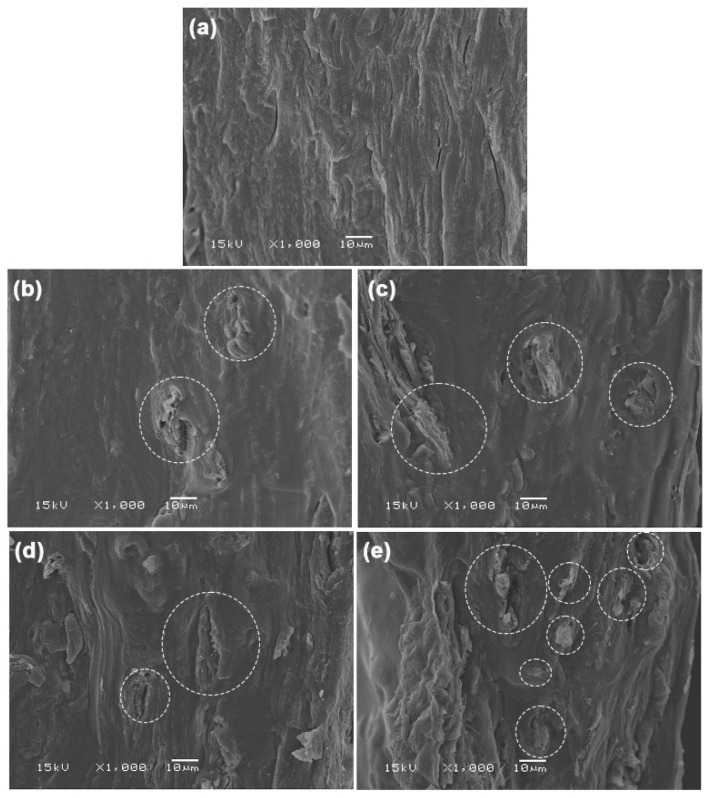
SEM images of cryo-fractured surfaces of glycerol-plasticized GG films: (**a**) without MCC and with MCC contents of (**b**) 5 wt%, (**c**) 10 wt%, (**d**) 20 wt%, and (**e**) 30 wt%. All films contain 30 wt% glycerol. Some MCC particles are indicated in white circles.

Figure S4 shows an SEM image of MCC powder. MCC has an irregular shape, with most particles consisting of cut cellulose fibers. Figure 12 displays SEM images of the cryo-fractured surfaces of MCC-reinforced and glycerol-plasticized GG films, alongside the glycerol-plasticized GG film for comparison. MCC particles were distinctly visible within the GG film matrices. Strong interfacial adhesion between the GG and MCC was evident. The high hydrophilic nature of both GG and MCC contributes to this observation. MCC has shown effective interfacial adhesion with various hydrophilic biopolymers, such as starch [24], TPS [25], and soy protein isolate [64], in earlier studies. The SEM results supported the strong interactions between the GG and MCC, which were further indicated by the previous analyses, including FTIR, TGA, and tensile tests. The advantageous interfacial adhesion between them augmented heat transfer, thereby enhancing the thermal stability of the GG film matrix. Additionally, it facilitated stress transfer, which in turn improved both the tensile strength and Young’s modulus of the GG film matrix.

#### 3.2.6. Moisture Content and Film Opacity

**Figure 13:**
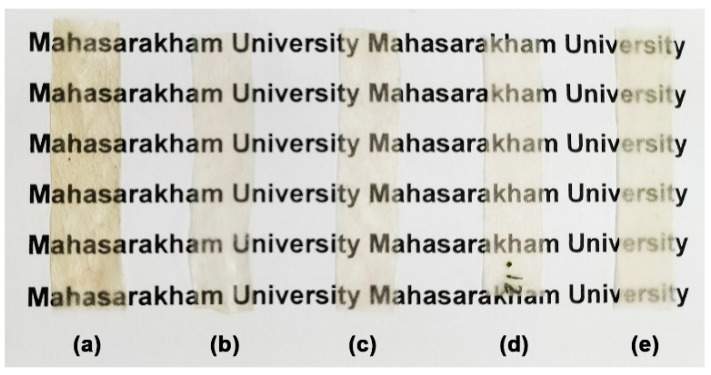
Photographs of thermo-compressed films of glycerol-plasticized GG: (**a**) without MCC and with MCC contents of (**b**) 5 wt%, (**c**) 10 wt%, (**d**) 20 wt%, and (**e**) 30 wt%. All films contain 30 wt% glycerol.

Table 6 presents the thickness, moisture content, and opacity of films containing varying amounts of MCC. The films became thicker as the amount of MCC in them went up. The addition of MCC may have increased the viscosity of the glycerol-plasticized GG, which could have led to reduced flow during the compression molding process. Rico et al. [25] reported that the addition of MCC increases the viscosity of the TPS in its molten state. The moisture content of the films consistently diminished with the increase in MCC content. This trend is consistent with research indicating that adding MCC decreases the moisture absorption of the films. The MCC filler’s higher degree of crystallinity contributes to this reduction [25,66]. The incorporation of additional MCC increased the opacity of the films. Figure 13 shows the appearance of glycerol-plasticized GG film, comparing the version without MCC (Figure 13a) to those with different MCC content levels (Figure 13b–e). As the amount of MCC increases, the film becomes increasingly opaque; however, the letters underneath the film remain visible. Packaging can use these films to maintain the visibility of product features.

## 4. Conclusions

The microcrystalline cellulose (MCC)-reinforced and glycerol-plasticized guar gum (GG) films were successfully produced using a thermo-compression process. The effects of glycerol and MCC on the properties of thermo-compressed GG films were examined in detail. The hydroxyl stretching bands shifted to lower wavenumbers from FTIR analysis, indicating hydrogen bond formation in the glycerol-plasticized GG films. Thermal stability of the GG film matrix improved with increased glycerol content, according to TGA analysis. XRD showed that glycerol addition enhanced crystallization of the GG film matrix. Elongation at break was higher, and tensile strength and Young’s modulus were lower in glycerol-plasticized GG films. This evidence suggests glycerol plasticizes GG film, increasing film flexibility. Glycerol increased moisture content in GG films. MCC hydrogen-bonded with the film matrix, improving tensile strength in glycerol-plasticized GG films. This procedure reduced the elongation at break of glycerol-plasticized GG films. MCC facilitates the thickening and opacification of the glycerol-plasticized GG films. The moisture content of the GG films decreased with the addition of MCC.

This study concluded that glycerol and MCC content can tailor the mechanical properties, hydrophilicity, and opacity of thermo-compressed GG films for packaging applications. We expect the research to enable conventional melt processing of biodegradable GG blends and composites. In the future, systematic research will examine thermo-compressed GG film biodegradation and barrier properties, such as the permeability of water vapor, oxygen, and carbon dioxide. Furthermore, improving the mechanical properties and water resistance of these GG films by blending them with other additives or polymers is also an intriguing prospect to further improve their suitability for alternative packaging applications.

## Figures and Tables

**Table 1:** TGA results of glycerol-plasticized GG films.

Sample Code	Glycerol Content (wt%)	Weight Loss of Moisture (50–120 °C) (%)	Char Residue at 800 °C (%)	*GG-T_max_* (°C)
GG	-	5.5	20.8	300
GG/15Gly	15	5.6	17.2	310
GG/30Gly	30	6.2	14.6	311
GG/45Gly	45	9.4	11.6	314

**Table 2:** Tensile properties of glycerol-plasticized GG films.

Sample Code	Glycerol Content (wt%)	Maximum Tensile Strength (MPa)	Elongation at Break (%)	Young’s Modulus (MPa)
GG	-	41 ± 3 ^a^	3 ± 1 ^a^	988 ± 16 ^a^
GG/15Gly	15	11 ± 1 ^b^	35 ± 4 ^b^	30 ± 5 ^b^
GG/30Gly	30	8 ± 1 ^c^	42 ± 5 ^c^	9 ± 2 ^c^
GG/45Gly	45	4 ± 1 ^d^	57 ± 5 ^d^	8 ± 2 ^c^

Values are presented as the mean ± standard deviation (*n* = 5). Column values denoted by the letters (^a^, ^b^, ^c^, and ^d^) exhibit significant differences (*p* < 0.05).

**Table 3:** Film thickness, moisture content, and film opacity of glycerol-plasticized GG films.

Sample Code	Glycerol Content (wt%)	Film Thickness (mm)	Moisture Content (%)	Film Opacity (mm^−1^)
GG	-	0.19 ± 0.06 ^a^	10.2 ± 0.2 ^a^	1.67 ± 0.09 ^a^
GG/15Gly	15	0.20 ± 0.05 ^a^	13.1 ± 0.4 ^a^	1.71 ± 0.08 ^a^
GG/30Gly	30	0.21 ± 0.06 ^a^	26.1 ± 0.6 ^b^	1.70 ± 0.10 ^a^
GG/45Gly	45	0.19 ± 0.08 ^a^	41.8 ± 0.5 ^c^	1.68 ± 0.12 ^a^

Values are presented as the mean ± standard deviation (*n* = 3). Column values denoted by the letters (^a^, ^b^, and ^c^) exhibit significant differences (*p* < 0.05).

**Table 4:** TGA results of MCC-reinforced and glycerol-plasticized GG films.

Sample Code	MCC Content (wt%)	Char Residue at 800 °C (%)	*GG-T_max_* (°C)	*MCC-T_max_* (°C)
GG/30Gly	-	14.6	310	-
GG/30Gly/5MCC	5	12.9	311	-
GG/30Gly/10MCC	10	13.1	311	355
GG/30Gly/20MCC	20	13.3	314	358
GG/30Gly/30MCC	30	13.2	314	358

**Table 5:** Tensile properties of MCC-reinforced and glycerol-plasticized GG films.

Sample Code	MCC Content (wt%)	Maximum Tensile Strength (MPa)	Elongation at Break (%)	Young’s Modulus (MPa)
GG/30Gly	-	8 ± 1 ^a^	42 ± 5 ^c^	9 ± 2 ^a^
GG/30Gly/5MCC	5	9 ± 1 ^a,b^	42 ± 4 ^b,c^	17 ± 2 ^b^
GG/30Gly/10MCC	10	10 ± 1 ^b,c^	39 ± 3 ^b^	26 ± 3 ^c^
GG/30Gly/20MCC	20	11 ± 1 ^c^	33 ± 3 ^a^	30 ± 2 ^c^
GG/30Gly/30MCC	30	16 ± 2 ^d^	32 ± 2 ^a^	67 ± 5 ^d^

Values are presented as the mean ± standard deviation (*n* = 5). Column values denoted by the letters (^a^, ^b^, ^c^, and ^d^) exhibit significant differences (*p* < 0.05).

**Table 6:** Film thickness, opacity, and moisture content of MCC-reinforced and glycerol-plasticized GG films.

Sample Code	MCC Content (wt%)	Film Thickness (mm)	Moisture Content (%)	Film Opacity (mm^−1^)
GG/30Gly	-	0.21 ± 0.06 ^a^	26.1 ± 0.6 ^c^	1.70 ± 0.10 ^a^
GG/30Gly/5MCC	5	0.34 ± 0.11 ^ab^	11.5 ± 1.2 ^b^	2.58 ± 0.11 ^b^
GG/30Gly/10MCC	10	0.43 ± 0.08 ^b^	10.1 ± 0.8 ^b^	3.04 ± 0.08 ^c^
GG/30Gly/20MCC	20	0.62 ± 0.10 ^c^	8.4 ± 0.9 ^a^	4.40 ± 0.06 ^d^
GG/30Gly/30MCC	30	0.83 ± 0.12 ^d^	8.1 ± 0.8 ^a^	5.07 ± 0.10 ^e^

Values are presented as the mean ± standard deviation (*n* = 3). Column values denoted by the letters (^a^, ^b^, ^c^, ^d^, and ^e^) exhibit significant differences (*p* < 0.05).

## Data Availability

The original contributions presented in this study are included in the article/supplementary material. Further inquiries can be directed to the corresponding authors.

## Supplementary Materials

The following supporting information can be downloaded at https://www.mdpi.com/article/10.3390/polym17233124/s1. Figure S1: ATR-FTIR spectra of (a) GG powder and (b) MCC.; Figure S2: TG and DTG thermograms of (a) GG powder and (b) MCC.; Figure S3: XRD patterns of (a) GG powder and (b) MCC. Figure S4: SEM image of MCC.
