# Supplementary material for: Preparation and Characterization of Thermo-Compressed Guar Gum/Microcrystalline Cellulose Composites for Applications in Sustainable Packaging
Source: Polymers (Basel). 2025 Nov 25;17(23):3124. doi: 10.3390/polym17233124 (PMC12694191; doi:10.3390/polym17233124)
Supplement: Supplementary file 1 [file polymers-17-03124-s001.zip › polymers-3998903-supplementary.pdf]

Supplementary materials

Preparation and characterization of thermo-compressed guar gum/microcrystalline cellulose composites for applications in sustainable packaging

Prasong Srihanam [1,*], Jenjira Jirum [1], Pakin Noppawan [2,3], Nuanchai Khotsaeng [4], and Yodthong Baimark [1,*]

1 Biodegradable Polymers Research Unit, Department of Chemistry and Centre of Excellence for Innovation in Chemistry, Faculty of Science, Mahasarakham University, Maha Sarakham 44150, Thailand; prasong.s@msu.ac.th (P.S.); jenjira.j@msu.ac.th (T. P.); yodthong.b@msu.ac.th (Y.B)
2 Department of Chemistry and Centre of Excellence for Innovation in Chemistry (PERCH-CIC), Faculty of Science, Mahasarakham University, Maha Sarakham 44150, Thailand; pakin.n@msu.ac.th (P.K.)
3 Sustainable Approaches for Materials, Agriculture, and Health Technology (SAMAHT) Research Unit, Mahasarakham University, Maha Sarakham 44150, Thailand; pakin.n@msu.ac.th (P.K.)
4 Faculty of Science and Health Technology, Kalasin University, Namon District, Kalasin 46230, Thailand; nuanchai.k@ksu.ac.th (N.K.)
* Correspondence: prasong.s@msu.ac.th (P.S.); yodthong.b@msu.ac.th (Y.B)

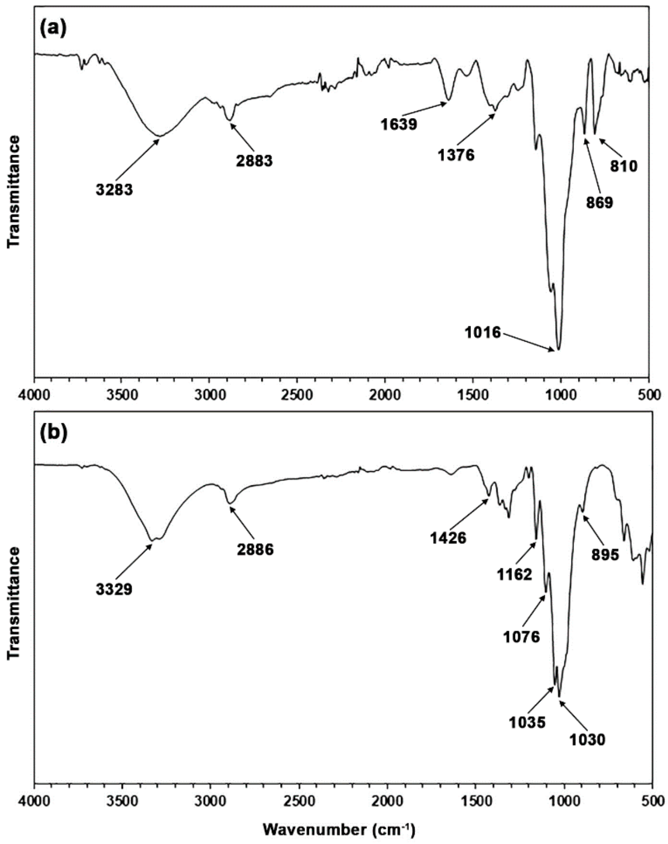

Figure S1. ATR-FTIR spectra of (a) GG powder and (b) MCC.

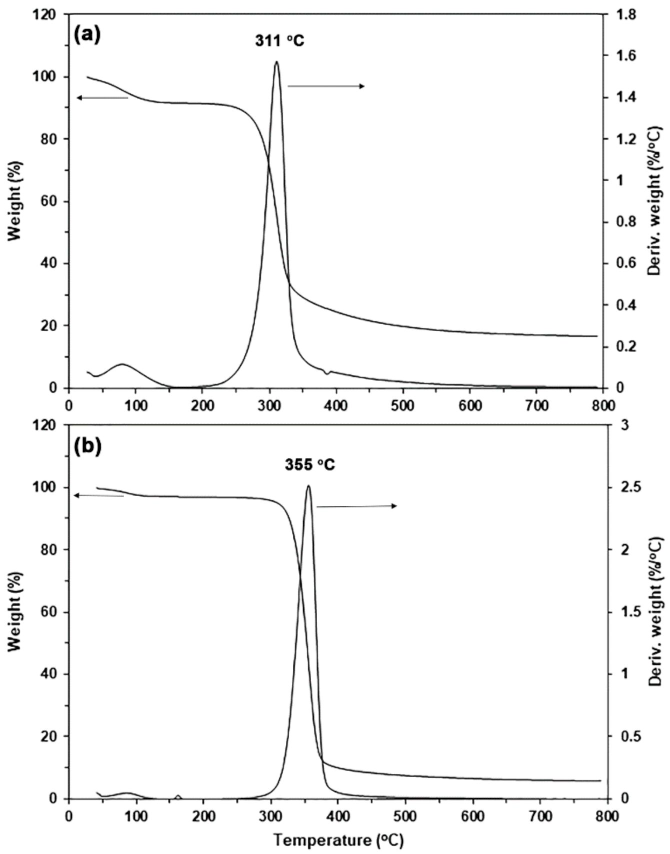

Figure S2. TG and DTG thermograms of (a) GG powder and (b) MCC.

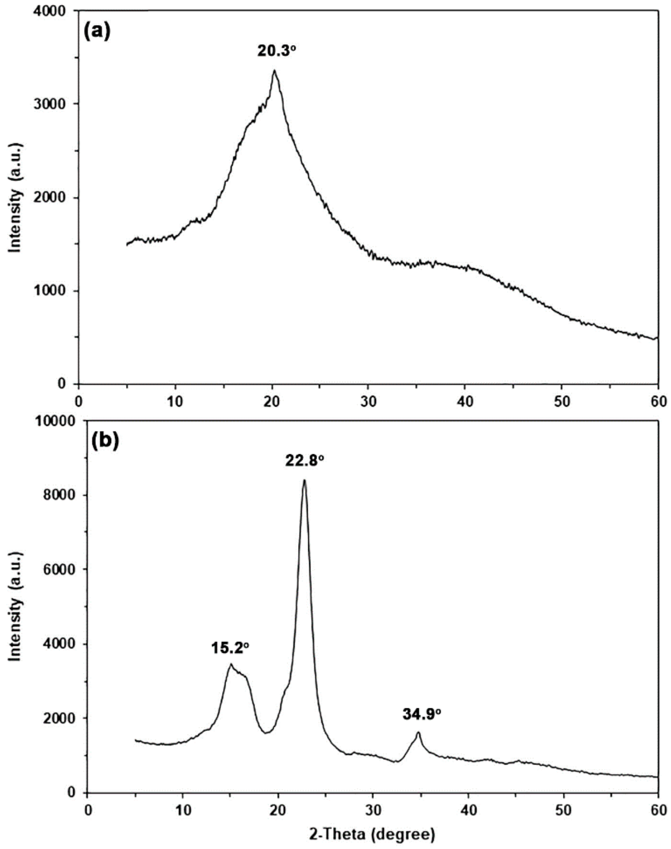

Figure S3. XRD patterns of (a) GG powder and (b) MCC.

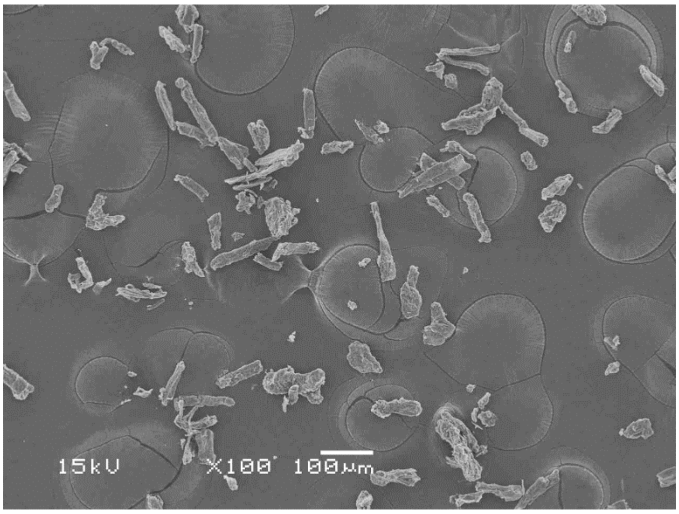

Figure S4. SEM image of MCC.
